# CRISPR/Cas9 uPAR Gene Knockout Results in Tumor Growth Inhibition, EGFR Downregulation and Induction of Stemness Markers in Melanoma and Colon Carcinoma Cell Lines

**DOI:** 10.3389/fonc.2021.663225

**Published:** 2021-05-14

**Authors:** Alessio Biagioni, Anastasia Chillà, Mario Del Rosso, Gabriella Fibbi, Francesca Scavone, Elena Andreucci, Silvia Peppicelli, Francesca Bianchini, Lido Calorini, Anna Li Santi, Pia Ragno, Francesca Margheri, Anna Laurenzana

**Affiliations:** ^1^ Department of Experimental and Clinical Biomedical Sciences “Mario Serio”, University of Florence, Firenze, Italy; ^2^ Department of Chemistry and Biology, University of Salerno, Fisciano, Italy

**Keywords:** urokinase-type plasminogen activator receptor, CRISPR, miR146a, melanoma, colon cancer

## Abstract

uPAR is a globular protein, tethered to the cell membrane by a GPI-anchor involved in several cancer-related properties and its overexpression commonly correlates with poor prognosis and metastasis. We investigated the consequences of uPAR irreversible loss in human melanoma and colon cancer cell lines, knocking out its expression by CRISPR/Cas9. We analyzed through flow cytometry, western blotting and qPCR, the modulation of the most known cancer stem cells-associated genes and the EGFR while we observed the proliferation rate exploiting 2D and 3D cellular models. We also generated uPAR “rescue” expression cell lines as well as we promoted the expression of only its 3’UTR to demonstrate the involvement of uPAR mRNA in tumor progression. Knocking out PLAUR, uPAR-encoding gene, we observed an inhibited growth ratio unexpectedly coupled with a significant percentage of cells acquiring a stem-like phenotype. *In vivo* experiments demonstrated that uPAR loss completely abrogates tumorigenesis despite the gained stem-like profile. Nonetheless, we proved that the reintroduction of the 3’UTR of PLAUR gene was sufficient to restore the wild-type status validating the hypothesis that such a region may act as a “molecular sponge”. In particular miR146a, by binding PLAUR 3’ UTR region might be responsible for uPAR-dependent inhibition of EGFR expression.

## Introduction

The urokinase plasminogen activator (uPA) receptor (uPAR) is a membrane receptor characterized by three globular domains, involved in several typical cancer features such as survival, invasion and migration, angiogenesis and intra-tumor recruitment of inflammatory cells (1–4). Commonly identified as a biomarker in breast cancer, the Plasminogen Activation system is validated for prognostic use in level-of-evidence-1 studies (5) and we recently identified uPAR as a potential marker in the acquisition of BRAF-I resistance in V600E mutant melanoma cells (6). Indeed, while uPAR is only tethered to the cell membrane by a GPI-anchor, lacking any intracellular domain, it is able to mediate connections with many other receptor systems such as receptor-tyrosine-kinases (EGFR, PDGFR), G-protein-coupled receptors and formyl peptide receptor-type 1 (7), activating such receptors even in absence of their specific ligand (8). In particular, the Epidermal Growth Factor Receptor (EGFR) is a fundamental partner of uPAR as it is able to transmit uPAR signal through the ERK pathway, generating a dynamic complex with the α_5_β_1_ integrin (9). Indeed, uPAR and EGFR are to date a well-known couple, able to regulate cancer cell proliferation, adhesion and migration (10). Recent studies also demonstrated that high levels of such molecules are associated with drug resistance in melanoma (6). Albeit this close association, little evidence has been reported about the cross-talk regulation although some studies demonstrated that uPAR may induce EGFR expression (11, 12). To better understand this mechanism, we exploited in the present study two melanoma cell lines, genetically identical but expressing different levels of uPAR and EGFR (6). Many studies, including our own, had focused on the features and the behavior of cancer cells after uPAR cleavage or downregulation, both *in vitro* and *in vivo*, using anti-uPAR oligodeoxynucleotide (ODN) (13, 14) and miRNA (15), exploiting uPAR inactivation specific cleavage systems such as MMP-12 (16), or inhibiting its interaction with the complex “uPAR interactome” by a specific uncoupler peptide (17). In one of our previous studies, we decided to exploit the CRISPR (Clustered Regularly Interspaced Short Palindromic Repeats)/Cas9 technique to establish two melanoma and one colon carcinoma cell lines with a complete uPAR KO (18), to better understand its role in tumor progression, examining the typical cancer hallmarks. This wide-spreading technology, based on a naturally-occurring system that protects bacteria from phages infections (19), is particularly useful being uPAR commonly modulated by many extracellular factors such as hypoxia, cytokines and transcription factors such as NF-kB and TCF/LEF (20) but also by cell-cell contact (21). Indeed, several inhibitors including small molecules, peptides and monoclonal antibodies, have been developed to block and inhibit uPAR function to study in deep how it might influence cancer progression. Moreover, other kinds of molecules were also established to inhibit its interaction with the integrins, the receptor-tyrosine-kinases and uPA. However, none of them have found application in clinical practice due to the poor affinity and bioavailability limit of such molecules. uPAR knockout *via* CRISP/Cas9 has already demonstrated promising results as its loss-of-function suppresses cell proliferation, migration and invasion in oral, colon carcinoma and neuroblastoma cell lines (22, 23). Being uPAR a master regulator of cancer proliferation the unbalanced activation of p38 and ERK due to the decreased activation of Akt might explain the induction of uPAR KO to a G0 state and thus to the incapacity to actively proliferate (18, 22). Moreover, uPAR loss led to decreased resistance to 5-FU, cisplatin, docetaxel, and doxorubicin demonstrating that it is also a major regulator of the drug resistance phenomenon (23). We previously identified in uPAR KO cells signs of mitochondrial biogenesis dysregulation and glycolysis inhibition paired with a more pronounced oxidative phosphorylation (OXPHOS) phenotype (18) that induced us to investigate if such features, commonly reported associated with cell plasticity (24, 25), might be also correlated with the presence of cancer stem cells (CSCs) markers. Indeed, while uPAR overexpression was associated for a long time with a stem-like phenotype (26, 27), it was also observed that its expression is completely absent in CD33+ myeloid precursors (high stem rank), CD14+ monocytic cells (a population that can differentiate into a host of different cells) and in CD3+ T lymphocytes and CD19+ B lymphocytes (28). Consequently, uPAR expression resulted increased only after granulocyte-colony-stimulating factor (G-CSF) treatment in CD33+ myeloid and CD14+ monocytic cells, while mobilized CD34+ hematopoietic stem cells remained uPAR negative. Therefore, uPAR plays a fundamental role as a differentiation antigen on cells of the myelomonocytic lineage and as an activation factor for monocytes and T lymphocytes (29). Such clues let us investigate whether uPAR deprivation in solid tumors may trigger the expression of Yamanaka’s factors (30), whose expression leads to the formation of induced pluripotent stem cells (iPSCs), and the appearance of stem-like surface markers. As reported above, uPAR lacks any intracellular domains, therefore its capability to regulated several so different pathways might be hidden in its transcript. Indeed, it was recently reported that PLAUR (uPAR encoding gene) 3’UTR might act as a molecular sponge, attracting and inhibiting many miRNAs. Through this complex mechanism it was demonstrated that uPAR, in a 3’UTR-dependent manner, may regulate several pro-tumoral factors, including Cathepsins and MMP2, TfR1, vimentin, ICAM-1, IL-8 and HGF in an acute leukemia cell model (31). In the present study we investigated all these aspects in the obtained uPAR KO clones, demonstrating *in vitro* and *in vivo* tumor growth inhibition coupled with unexpected features, sometimes unrelated to what previously reported by using short-term silencing methods, such as appearance of stemness markers and loss of EGFR.

## Materials and Methods

### Cell Lines

Human melanoma A375p (CRL­1619) and colon cancer HCT 116 (CCL­247) cell lines were obtained from American Type Culture Collection while human melanoma cell line A375-M6 was isolated from a lung metastasis of SCID bg/bg mice i.v. injected with A375p (32) and validated through STR profile (BMR Genomics). All the cell lines were grown in DMEM with 10% FBS (Euroclone, Milano, Italy). uPAR KO and “rescue expression” cell clones were the same obtained in our previous work and were characterized and validated as previously described (18). Cells were tested every two weeks for Mycoplasma by PCR using two universal primers (MGSO and GPO1) (33).

### Transfection and Plasmid

uPAR KO cells were obtained as previously reported (18). Briefly, the plasmids (sc-400666-NIC) for CRISPR/Cas9, targeting PLAUR exon 3, were obtained from Santa Cruz Biotechnology (Santa Cruz, California, USA) and transfected according to the manufacturer’s instructions. sgRNA were however subjected to off-target site analysis throughout the Cas-OFF Finder software (http://www.rgenome.net/cas-offinder/). No off-target sites, in the loci of the most likely off-target activity of the CRISPR/Cas9 system targeted by the chosen sgRNAs, could be detected. Cells were then sorted for GFP marker and selected with 1 µg/ml puromycin for 2–3 weeks (Sigma-Aldrich, Saint Louis, Missouri, USA), singularly characterized using Western Blotting, qPCR and PCR on the full-length mRNA. For the uPAR rescue expression experiment, cells were stably transfected using an Okayama-Berg vector containing uPAR cDNA (including its 3’UTR) and selected with G418 as previously reported (34). The plasmid encoding PLAUR 3’ UTR was kindly provided by Prof. Ragno Pia (31).

### RNA Extraction and Quantitative PCR

Total RNA was prepared using Tri Reagent (Sigma-Aldrich), agarose gel-checked for integrity, and reverse transcribed (from 500 ng to 1 µg measured through Thermo Fisher Scientific NanoDropOne) with cDNA synthesis kit (BioRad, Milano, Italy) according to the manufacturer’s instructions. Selected genes were evaluated by Real-Time RT-PCR (qPCR) using SsoAdvanced Universal Green Mix (BioRad) with BioRad CFX96 qPCR System (BioRad) with the reported amplification steps: polymerase activation 95°C for 3 min, denaturation 95°C for 10 s and annealing/extension 60°C for 30 s (the last two steps repeated for 40 cycles). Melt curve analysis was evaluated every qPCR performed following the in-built Biorad CFX96 protocol. Fold change was determined by the comparative Ct method using β2-Microglobulin and 18S as normalization genes. Primer sequences (IDT, TemaRicerca, Bologna, Italy) are reported in **Table 1**.

**Table 1:** List of all the primers used.

Gene	Sense	Antisense
***B2M***	GCCGTGTGAACCATGTGACT	GCTTACATGTCTCGATCCCACTT
***18S***	CCAGTAAGTGCGGGTCATAAG	GCCTCACATAA-CCATCCAATC
***PLAUR***	GCCCAATCCTGGAGCTTGA	TCCCCTTGCAGCTGTAACACT
***3’UTR***	ACCTGAAATCCCCCTCTCTG	CCACTGGTACAAAATCTTTATGTAAG
***KLF-4***	GCAGCCACCTGGCGAGTCTG	CCGCCAGCGGTTATTCGGGG
***Nanog***	ACCTTGGCTGCCGTCTCTGG	AGCAAAGCCTCCCAATCCCAAACA
***Oct3/4***	TTTTGGTACCCCAGGCTATG	GCAGGCACCTCAGTTTGAAT
***SOX2***	GAGCTTTGCAGGAAGTTTGC	GCAAGAAGCCTCTCCTGAA
***c-myc***	AATGAAAAGGCCCCAAGGTAGTTAT	GTCGTTTCCGCAACAAGTCCTCTTC
***MGSO***	TGCACCATCTGTCACTCTGTTAACCTC	
***GPO1***	ACTCCTACGGGAGGCAGCAGTA	

### miR146a Bioavailability Inhibition

miR146a activity was inhibited by using simultaneously two anti-miR146a, targeting the 3p and the 5p (Thermo Fisher Scientific). Such anti-miRNAs were transfected into the cell lines through Lipofectamine 3000 (Thermo Fisher Scientific) according to the manufacturer’s instruction. According to our optimized conditions, a final concentration of 30 nM was enough to inhibit miR146a activity for at least 72 h. Higher concentrations (a maximum of 100 nM was tested) did not exert better or long-lasting effects.

### Flow Cytometry Analysis

Cells were harvested with Accutase (Euroclone), washed once with cold PBS and then stained with fluorochrome-conjugated mAbs anti-CD44 (Immunotools GmbH, Germany), -CD133, -CD243 and -EGFR (eBioscience, Milano, Italy), and ALDH1 (Abcam, Milano, Italy) for 1 h on ice in dark. Irrelevant fluorochrome-conjugated IgG was used in all experiments as a negative control. Cells were analyzed by flow cytometry BD-FACS Canto II coupled with DivaSoftware (BD Biosciences, Milano, Italy) while statistical analysis was supported by FlowJo software (LLC, BD Biosciences).

### CellTrace CFSE Proliferation Assay

Cells were harvested with Accutase (Euroclone) and stained with CellTrace CFSE (Thermo Fisher Scientific) according to the manufacturer’s instruction. Cells were harvested 24 and 48 h after the start of the experiment and compared with control (T0). Cells were then fixed and analyzed by flow cytometer through ModFitLT software (BD Biosciences).

### Tumor Spheroid Formation

Tumor cell monolayers were harvested and 200 μl/well of cell suspensions (0.5 × 10^4^ cells per well) were dispensed into a 96-well flat-bottomed plate pre-coated with 1.5% Agar as previously described (35) using a multichannel pipette. Plates were incubated for 4 days at 37°C, 5% CO2, 95% humidity. We visually confirmed tumor spheroid formation and images were taken at regular intervals. The radius of each spheroid was used to calculate the volume (μm^3^): V = 4/3 π r^3^

### *In Vivo* Tumor Proliferation

All *in vivo* procedures were approved by the ethical committee of the Animal Welfare Office of the Italian Work Ministry and conformed to the legal mandates and Italian guidelines for the care and maintenance of laboratory animals (Auth. N° 401/2015-PR). Six- to eight-week-old female NOD SCID (Charles River, Lodi, Italy) were injected into the flanks with 1.0 × 10^6^ cells (n = 5). To determine tumor volume, the greatest longitudinal diameter (length) and the greatest transverse diameter (width) were determined with an external caliper. Tumor volume was calculated by the following formula: tumor volume = length × width2 × 0.5. Mice were monitored every two days and sacrificed before showing evident physical signs of discomfort with an overdose of isoflurane.

### *In Silico* miRNAs Analysis

The analysis of all the possible miRNAs targeting the PLAUR 3’UTR region was retrieved by TargetScan software (36), which predicts miRNA target genes by searching for the presence of six to eight mer sites that match the seed region of a given miRNA and make alignment to all mammals or vertebrates conserved sites.

### *In Vitro* Limiting Dilution Assay

Control or uPAR KO cells were seeded into ultra-low attachment 96-well plate at different cell doses, with a maximum of 100 cells per well and a minimum of one cell per well, and incubated in DMEM/F12 supplemented with N2, 20 µg/ml insulin, 20 ng/ml FGF-2, and 20 ng/ml EGF (provided by Thermo Fisher Scientific) at 37°C. Colony formation was assessed by visual inspection. For each dilution series, we counted wells that showed sphere formation on day 11. Data were analyzed and displayed using the Extreme limiting dilution assay (ELDA) software available at http://bioinf.wehi.edu.au/software/elda/ (37).

### Western Blot Analysis

Cells were lysed in RIPA buffer (Merck Millipore, Milano, Italy) containing PMSF (Sigma-Aldrich), sodium orthovanadate (Sigma-Aldrich), and protease inhibitor cocktail (Calbiochem, San Diego, CA, USA), sonicated and centrifuged 15 min at 14,000 rpm at 4°C. 50 µg of protein, evaluated through the BCA method, were separated on Bolt^®^ Bis-Tris Plus gels, 4–12% precast polyacrylamide gels (Thermo Fisher Scientific). Fractionated proteins were transferred to a PVDF membrane using the iBlot 2 System (Thermo Fisher Scientific). Following 1-h blocking with Odyssey blocking buffer (Bioclass, Pistoia, Italy), the membrane was probed overnight at 4°C with the respective primary antibody. Primary antibodies used were: anti-Nanog (1:500) provided by Genetex (CA, USA), anti-cMyc (1:1,000) and anti-α-Tubulin (1:1,000) provided by Cell Signaling, anti-EGFR (1:500) provided by Santa Cruz Biotechnology. Protein bands were analyzed by Odyssey Infrared Imaging System (Licor Bioscience) using ImageJ software (developed by Wayne Rasband, National Institutes of Health, Bethesda, MD, USA) for protein quantification.

### Cell Viability Assay

Cell viability and death percentage were determined by flow cytometer using Annexin V FITC-conjugated (Immunotools GmbH, Germany) and PI (Sigma-Aldrich) according to the manufacturer’s protocol. Briefly, cells were harvested with Accutase (Eurolone), collected in flow cytometer tubes (1 × 10^5^ cells/tube), washed once in PBS and incubated 15 min at 4°C in dark with 100 µl Annexin Binding buffer (100 mM HEPES, 140 mM NaCl, 25 mM CaCl_2_, pH 7.4), 1 µl of 100 µg/ml PI working solution, and 5 µl Annexin V FITC-conjugated. The samples were then analyzed at BD FACSCanto II. Cellular distribution depending on Annexin V and/or PI positivity allowed the measurement of the percentage of viable (Annexin V and PI-negative cells) and death cells (Annexin V and/or PI positive cells).

### Statistics

Results are expressed as means ± SD. Multiple comparisons were performed by the Student test or One-way ANOVA using GraphPad Prism 6. Statistical significances were accepted at p <0.05.

## Results

### Loss of uPAR Causes Significant Growth Inhibition

**Figure 1:**
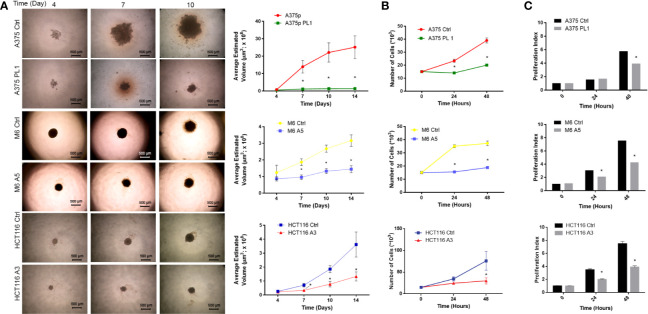
**(A)** Agar-coated 96-well flat-bottomed plates were used to generate spheroids (a single spheroid per well). Starting from day 4 post generation, images were obtained at intervals using an inverted microscope. The analysis was carried out using ImageJ software and growth curves were obtained (n = 60 spheroids/timepoint; magnification 4×, bar 500 µm). **(B)** Cellular growth counting the total number of cells 24 and 48 h after the initiation of the culture. (n = 3). **(C)** Proliferation Index: fold expansion during culture (ratio of final cell count to starting cell count) as defined in ModFitLT (n = 5). Values are mean ± SD *p <0.001 (Student’s Test).

Starting from previous observations about the decreased glycolytic capacity in absence of uPAR (18, 38), we focused on the analysis of cell proliferation. To accomplish such aim, we used previously obtained and selected individual clones (18), after CRISPR-mediated uPAR KO, which were called A375 PL1 from A375p, M6 A5 from A375M6 (melanoma cell lines) and HCT116 A3 from HCT116 (colon carcinoma cell line). Control cells were instead obtained through transfection with a plasmid containing a scramble sgRNA. Thus, we decided to generate spheroids of uPAR KO and control cells, thereby testing in such a way the tumor growth with a method that most closely mimics the tumor growth *in vivo*. While M6 and HCT116 arrange themselves in tight spheroids, A375p formed the so-called “Loose Aggregate Spheroids” probably due to their melanocytic origin, as they grow as a compact mass at the center with friable aggregate cells all around (35). We monitored them for 14 days and observed evident growth inhibition in all uPAR KO cells (**Figure 1A**). We repeated the assay in 2D culture by counting cells at 24 and 48 h, obtaining similar results (**Figure 1B**). We further confirmed such data by determining the proliferation index using CFSE labeling dye (**Figure 1C**). As shown in **Figure 1C**, no evident changes occurred at 24 h for A375 PL1, probably due to the latency phase of these cells, but at 48 hours we observed a decreased proliferation index (the average number of divisions that all responding cells have undergone since the initiation of the culture). Although the demonstrated different proliferation rates between control and uPAR KO cells, we did not detect any significant change in cell viability (**Supplementary Figure 1**).

### *In Vivo* uPAR KO Growth Inhibition

**Figure 2:**
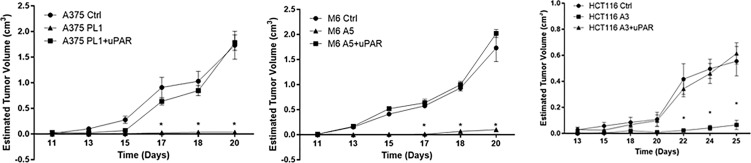
Tumor growth curves of A375 (left), M6 (center) and HCT116 (right); from tumors appearance, masses were measured every two days with a caliper. Each point represents the mean tumor volume (± SD) of measurements from two tumors for every single mouse (n = 5). *p <0.001 (One-Way ANOVA).

To further confirm that loss of uPAR led to the inhibition of tumor growth, we exploited “uPAR rescued expression” cell lines, previously obtained (18), briefly forcing the re-expression of uPAR by stably transfecting KO cells with a plasmid containing the sequence of PLAUR gene. Thus, we inoculated subcutaneously in NOD SCID mice, Control, uPAR KO and “rescued expression” cells. As shown in **Figure 2**, after about 20–25 days from the inoculation, Control and uPAR rescue groups mice required sacrifice, due to excessive tumor dimensions, while we demonstrated significant growth inhibition in M6 A5 and HCT116 A3 and absolutely no growth in A375 PL1, confirming *in vivo* what we previously observed *in vitro*.

### uPAR Deficiency Induces the Acquisition of a Stem-Like Profile

**Figure 3:**
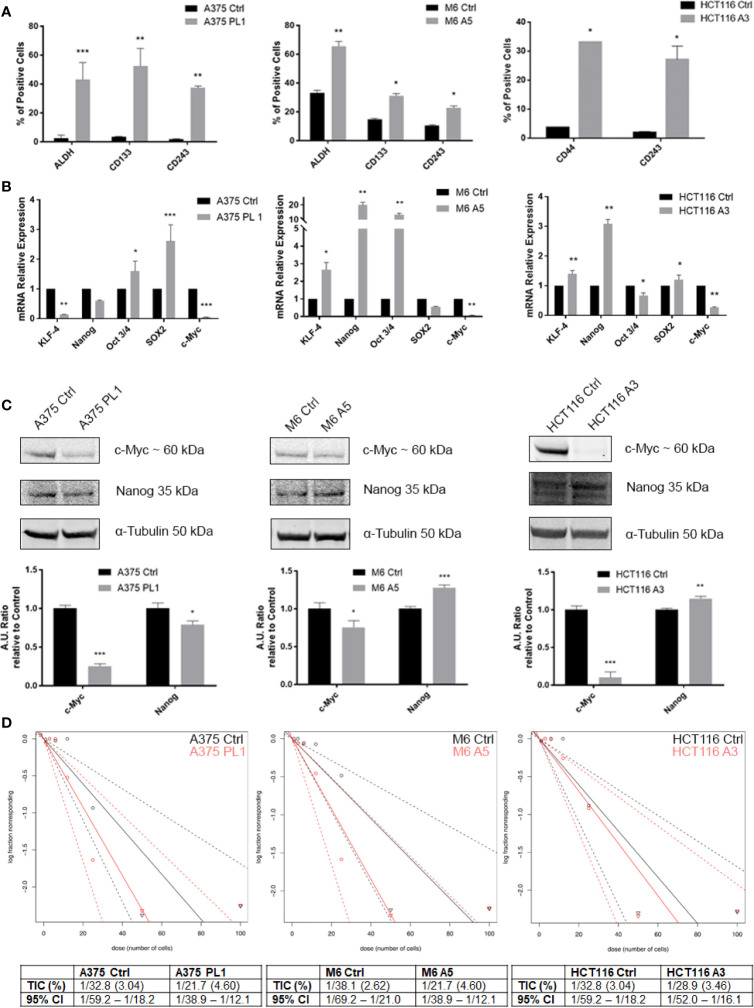
**(A)** Melanoma uPAR KO cells were tested for ALDHA1 and CD133 while colon carcinoma uPAR KO cells were tested for CD44 by FACS analysis. CD243 expression was evaluated on all the three uPAR KO cell lines (n = 3). **(B)** Total RNA isolated using Tri Reagent was subjected to RT–PCR and qPCR was performed (n = 3). **(C)** Whole-cell lysates were analyzed by Western Blot for c-Myc, and Nanog expression and α-Tubulin was used as a loading control (n = 3). **(D)** Limiting dilution sphere assay of uPAR KO and control cells. ELDA analysis plot (upper) and relative table reporting the TIC percentage (lower) (n = 5). Values are mean ± SD; *p <0.01, **p <0.001, ***p <0.0001 (Student’s Test).

Given the low proliferation rate and our previous reported evidence about the relationship between uPAR loss and the acquisition of a more oxidative metabolic phenotype and the dysregulated mitochondrial biogenesis (18), we decided to investigate if such features might be correlated with a stem-like phenotype of the uPAR KO cells, being such features commonly associated to enhanced cell plasticity (24, 25). Even if a universal marker for Cancer Stem Cells (CSCs) identification remains undiscovered, CSCs often express distinctive markers like CD133, CD44, ABCB1/5 (CD243), ALDH1 and many others, though many of them are tissue- and tumor-related (39, 40). As shown in **Figure 3A**, by flow cytometer analysis, we observed that uPAR KO cell lines, kept in standard culture media and conditions, express stem cell markers, otherwise poorly or not expressed at all in Control cells. Indeed, A375p PL1 and M6 A5 have an increased expression level of ALDH1 and CD133 while HCT116 A3 upregulated CD44. Moreover, also CD243 is expressed in all uPAR negative cells. ALDH1 is a common well-known marker for many types of tumors (41), while CD133 and CD44 are reported to be closely associated with both melanoma and colon carcinoma (42–46). To further analyze uPAR-mediated stem-like phenotype acquisition, we performed a qPCR analysis for the Yamanaka’s factors (30) and Nanog, one of the genes involved in the maintenance of the stemness state (47, 48). As shown in **Figure 3B** while c-Myc is substantially not expressed in all the three KO cell lines, according to the observed slower proliferation rate (49, 50), all the other stem markers are upregulated in M6 A5 and HCT116 A3 except SOX2 and Oct3/4 respectively. In A375 PL1 we observed instead an overexpression of Oct3/4 and SOX2 and a downregulation of KLF-4 while Nanog was not significantly changed. We were able to validate through Western Blot the expression of c-Myc and Nanog as shown in **Figure 3C**. Moreover, being the self-renewal potential one of the hallmarks of CSCs, we decided to test *in vitro* the potential of the evaluated cell lines to form tumor-spheres using a limiting dilution assay, evaluating sphere formation 10 days later cell plating in a typical stem-selecting media (51). By analyzing the results *via* ELDA software, we assessed that all uPAR KO cells demonstrated an increased generation of spheres to controls, resulting in a significant increase in their tumor-initiating cells (TIC) percentage (**Figure 3D**) (37). However, due to the extreme variability of the expression of the transcripts among the cell line models included in our study, probably caused by a complex cells heterogeneity as yet reported (52–58), we decided to perform further analysis taking into consideration the surface stem cell markers which were found to be more reliable.

### PLAUR 3’UTR Is Involved in CSCs Pattern Acquisition

**Figure 4:**
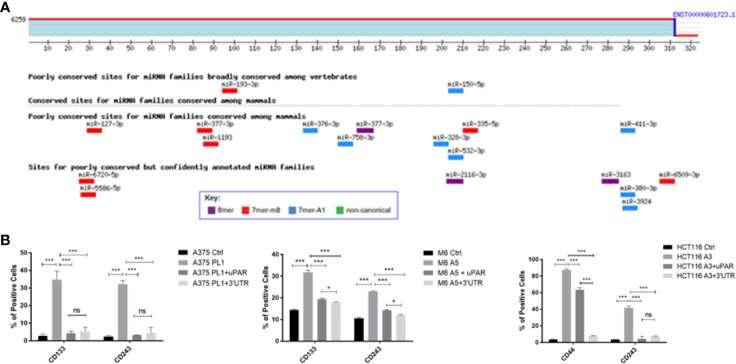
**(A)** PLAUR 3’UTR was subjected to bioinformatical analysis *via* TargetScan software, to identify probable interacting miRNAs candidates and their binding sites. **(B)** uPAR KO cells were tested for CD133 while colon carcinoma uPAR KO cells were tested for CD44 by FACS analysis. CD243 expression was evaluated on all the three uPAR KO cell lines (n = 3). Values are mean ± SD; *p <0.01, ***p <0.0001, ns, not significant (One-Way ANOVA).

To better understand how uPAR loss may be involved in the induction of a stem-like profile we decided to perform an antigenic surface analysis not only on uPAR KO and rescue expression cells but also adding uPAR KO cells stably transfected with a plasmid containing only PLAUR 3’UTR (**Supplementary Figure 2**). Indeed, PLAUR 3’UTR was recently reported (31) to behave as a molecular sponge, attracting many miRNAs and thus inactivating them. As shown in **Figure 4A** we performed an *in silico* analysis through TargetScan software, looking for the six to eight mer sites that matched the seed region of a given miRNA and aligning them to all mammals or vertebrates conserved sites (36). Analyzing the different miRNAs with a probable affinity for PLAUR 3’UTR is not clear from the current literature if some of them might be responsible for the unexpected acquisition of stem-like markers (**Table 2**). However, analyzing the previously reported stem surface antigens (**Figure 4B**), we demonstrated an almost complete restoration of the expression of CD133 in the two melanoma cell lines, CD44 in the colon carcinoma and CD243 in all the three cell lines to control levels, after the reintroduction of the complete uPAR sequence or only its 3’UTR, proving that providing back PLAUR 3’UTR is sufficient to revert the expression of the stem-related markers. Actually, we also need to point out that ALDH is the only marker that did not show any changes after uPAR reintroduction (in its entire form or only the 3’UTR) (Data not shown).

**Table 2:** Analysis of predicted miRNAs.

miRNA	Target	Reference
**miR-193-3p**	TGFBR3	(59)
**miR-150-5p**	VEGFA/EGR2/c-Myb	(60–62)
**miR-127-3p**	KMT5a/CISD1	(63, 64)
**miR-377-3p**	GSK‐3β/JAG1/XIAP/ZEB2	(65–67)
**miR-1193**	TM9SF3	(68)
**miR-376-3p**	FGFR1/ARID2/SYF2/CCND1	(69–72)
**miR-758-3p**	MDM2/mTOR/CBX5	(73, 74)
**miR-335-5p**	CCNB2/MAPK10/LDHB/TPX2	(75–78)
**miR-328-3p**	Girdin/ERMP1/MMP9/DDB2/MMP16	(79–83)
**miR-532-3p**	PTPRT/CTNNB1/ETS1/CCR7/FOXP3	(84–89)
**miR-411-3p**	Smurf2	(90)
**miR-6720-5p**	Unknown	
**miR-5586-5p**	Unknown	
**miR-2116-3p**	MYC	(91)
**miR-3163**	ADAM-17/Skp2	(92, 93)
**miR-6509-3p**	GAS1 (probable)	(94)
**miR-380-3p**	SOX6/FOXO1/Nrf2	(95–97)
**miR-3924**	WNT5A	(98)

### miR146a Is Responsible for uPAR-Dependent Inhibition of EGFR Expression

**Figure 5:**
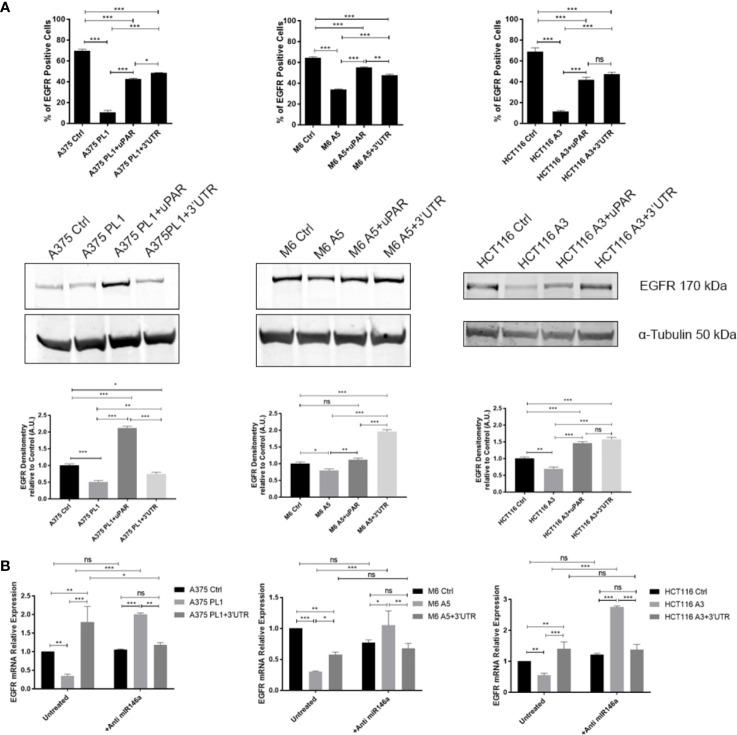
**(A)** Cells were tested for EGFR expression by FACS analysis and Western Blotting (n = 3). **(B)** Total RNA isolated using Tri Reagent was subjected to RT–PCR and qPCR, evaluating EGFR expression after anti-miR146a treatment for 24 h (n = 3). Values are mean ± SD; *p <0.01, **p <0.001, ***p <0.0001, ns, not significant (One-Way ANOVA).

To validate if such a mechanism might be responsible for the unpredicted behavioral changes in uPAR KO cells, we decided to analyze miR146a, which was recently experimentally identified to be bound on PLAUR 3’ UTR (31), albeit not shown in the previous analysis in **Figure 4A**, and it is commonly involved in the regulation of EGFR expression (99, 100). Indeed, as shown in **Figure 5**, we evaluated EGFR expression by flow cytometry and Western Blotting (a) observing a strong downregulation in all uPAR KO cells which is in turn reverted by the reintroduction of uPAR full length or uPAR-3’UTR, reflecting the above-reported behavior. To evaluate if miR146a might be responsible for such a mechanism we exploited a specific miR-inhibitor, composed of a mix of 3p and 5p anti-miR146a oligonucleotides, in order to block its functionality. As shown in **Figure 5B**, EGFR expression was evaluated by qPCR after 24 h of treatment with the anti-miR146a, demonstrating that in all uPAR KO cell lines EGFR expression levels were strongly increased. Such a shift in EGFR expression after the blockade of miR146a activity was also evaluated and validated by flow cytometry (**Supplementary Figure 3**).

## Discussion

Here we have exploited three uPAR KO cell lines, *via* a CRISPR/Cas9 approach, to investigate the consequences of its total loss in tumor progression. uPAR KO cells exhibited a lower proliferation rate as expected (22) since uPAR plays an important role in the stimulation of the PI3K/Akt and MAPK axis (6, 20, 38, 101) (**Supplementary Figure 4**). We observed such diminished proliferation both *in vitro*, exploiting canonical 2D and 3D cell model techniques, and *in vivo*, through the subcutaneous injection of uPAR KO cancer cells and cells that regained PLAUR expression *via* stable transfection with a plasmid bearing its full-length form (including the 3’UTR region). Our previous studies, focused on the metabolic changes in uPAR KO cells, evidenced that uPAR loss led to mitochondrial biogenesis deregulation and the inhibition of the glycolytic pathway (18, 38). Such clues, i.e. low proliferation rate, OXPHOS predominant metabolism paired with mitochondrial biogenesis deregulation and high mitochondrial membrane potential, are all typical features of cancer stem cells and thus led us to investigate if uPAR KO cells may have gained a stem-like profile (102–104). Indeed, uPAR KO cell lines express stem-like cell markers otherwise not or partially expressed in control cells. The analysis of Yamanaka’s factors (30) revealed a confusing pattern as the only concordant gene which is not expressed in all the three cell lines is c-myc, according to the slower proliferation rate (49, 50). The discordant data about KLF-4 and Oct3/4 expression can be due to the fact that, besides controlling cancer cell reprogramming (105), they also regulate the epithelial-to-mesenchymal transition, modulating phenomena such as migration and proliferation (106). Moreover, Nanog is reported to play a fundamental role mainly in the maintenance of the stem-like status (48). Analyzing the stem-related markers *via* flow cytometer, we evaluated that, A375p PL1 and M6 A5 over-expressed ALDHA1 and CD133 while HCT116 A3 up-regulated CD44. CD243, involved in multidrug resistance, is over-expressed in all uPAR negative cells. Moreover, using the ELDA analysis software we also observed an increased self-renewal capability of all the uPAR KO cells. These results are not in agreement with the previous literature (23, 107), not complying with ongoing beliefs on uPAR functions, that directed historical lines of research, included our own and will need further investigations analyzing more stem-related markers and exploiting other *in vitro* and *in vivo* functional assays. While previous researches focused on the blockade of uPAR functions *via* peptides or through short-term silencing, in this work we permanently eradicated the entire PLAUR mRNA expression by CRISPR/Cas9, leading to the loss-of-function of all the untranslated regions of its transcript. Importantly, restoring uPAR full-length expression or even only transducing its 3’UTR sequence, the stem-like status was reverted. We need to point out that for “uPAR rescue experiments”, we decided to exploit an “old fashioned” Okayama-Berg plasmid expressing a full-length cDNA uPAR, which also includes the 3’UTR as reported by Roldan et al. (108). Moreover, as demonstrated by Debeb et al. and Montuori et al., HEK293T, which is a human embryonal kidney-derived cell line commonly used for adeno- and lentiviral particles preparation, is characterized by stem markers such as ALDHA1 and CD44, but does not express uPAR (109, 110). Taken together, such data support the idea that the partial stem status induced by loss of uPAR in cancer cells may confer to such cells the characteristics required to give origin to the minimal residual disease (111). The main features of such cancer stem cells must be the maintenance of the stem state, resistance to chemotherapy and quiescence, all characteristics that define a high hierarchical stemness grade. The re-activation of quiescent CSCs is likely involved in tumor relapse, an event that may occur even decades after disease remission (112, 113). Our data suggest that in the absence of uPAR, a reactivation of cell proliferation is impossible even for CSCs and further experiments using fine reintroduction of uPAR at a genomic level controlled by a physiologic promoter are required to validate that uPAR expression might be one of the stem compartment controllers. Indeed, as demonstrated *in vitro* and *in vivo*, uPAR is mandatory for cell proliferation and therefore its permanent loss does not allow to proper study many stem features, as cells are incapable to demonstrated their tumorigenic potential despite the stem-like phenotype. This assumption is further sustained by the strong EGFR reduction that parallels uPAR KO and leads to a non-proliferative state. Indeed, EGFR plays an important role in the cell cycle and thus its decrease could likely lead cells to a G0 state, meaning a drastic proliferation reduction as reported by Lui et al. (114). The relationship between EGFR and cell cycle has been primarily elucidated by examining the effects of specific EGFR-targeting agents on cancer cells: attenuation of EGFR growth signaling by various therapeutic agents (i.e. EGFR antisense, monoclonal antibodies against EGFR, or specific tyrosine kinase inhibitors) results in cell cycle arrest in many tumor systems (115, 116). Moreover, EGFR regulates MYC triggering the activation of the Ras/Raf/MEK/ERK pathway and the PI3K-Akt axis (117). ERK activation induces cell proliferation through transactivation of the cyclin D1 gene and c-myc (118). Again, no evidence of a parallel reduction of EGFR following uPAR KO or knockdown was previously reported. In our previous works using uPAR clearing methods such as anti-uPAR ODN (19, 20) and miRNA (21), or exploiting uPAR inactivation specific cleavage systems such as MMP-12 (28) or uncoupling agents (17), we have never observed the induction of a stem-like profile. The CRISPR/Cas9 approach is deeply different from the previous ones because it acts on a genomic level eradicating from the cell not only the protein but also the mRNA. PLAUR mRNA has been reported (31) to bear a 3’UTR sequence which may act as a molecular sponge for several miRNAs, that are incapable to perform their actions when the uPAR transcript is strongly expressed. uPAR loss may trigger the release of such miRNAs inducing an undifferentiation process toward a more staminal status. Being CSCs, a topic still not fully understood and with many unclear molecular mechanisms, we were not able at the current status to identify which miRNAs, released by uPAR loss, may have triggered such stem-like conversion. From the analysis performed on the probable miRNAs with an affinity for PLAUR 3’UTR, only miR-328-3p was reported to play stem-related functions maintaining CSC properties in ovarian cancer and enhancing metastasis *via* the downregulation of the DNA damage binding protein 2 (DDB2). Moreover, it is important to note that miR-6720-5p and miR-5586-5p are to date completely unknown regarding their function and targets. However, it was sufficient to provide to uPAR KO cells an anti-miR146a to revert EFGR expression, almost to controls level. Indeed, it is well known that miR146a is a master controller of EGFR expression in cancer cells (99, 100). With our data, we were able to validate and prove for the first time in solid tumors, that uPAR 3’UTR might function as a molecular sponge attracting miRNAs which were consequently released by uPAR loss. We also evidenced that uPAR KO mediated by CRISPR/Cas9 is able to induce a stem-like phenotype in melanoma and colon carcinoma cells, overexpressing stem-related antigens and transcription factors and enhancing their self-renewal capabilities. Finally, we were able to observe that the molecular sponge mechanism, controlling miR146a, is responsible for uPAR-mediated EGFR expression.

## Data Availability Statement

The original contributions presented in the study are included in the article/**Supplementary Material**. Further inquiries can be directed to the corresponding authors.

## Ethics Statement

The animal study was reviewed and approved by Animal Welfare Office of the Italian Work Ministry (Auth. N° 401/2015-PR).

## Author Contributions

Conceptualization, AB, MR, GF, and AL. Funding acquisition, MR, LC, and GF. Investigation, AB, EA, SP, FB, AC, FS, and FM. Resources, ALS and PR. Methodology, AB and AL. Supervision, AC and AL. Validation, AL, EA, and SP. Writing—original draft, AB, MR, FM, AC, and AL. All authors contributed to the article and approved the submitted version.

## Funding

This work was supported by Ente Cassa di Risparmio di Firenze (2016.1225) and Associazione Italiana per la Ricerca sul Cancro (IG 2013 N. 14266). AB and EA were supported by a post-doctoral fellowship of the Fondazione Italiana per la Ricerca sul Cancro (FIRC). AC was a recipient for a Global Marie Curie Fellowship.

## Conflict of Interest

The authors declare that the research was conducted in the absence of any commercial or financial relationships that could be construed as a potential conflict of interest.

The reviewer MT declared a shared affiliation with several of the authors ALS and PR to the handling editor at time of the review.

## Supplementary Material

Supplementary Figure 1 Annexin V/PI assay on control and uPAR KO cells. Values are mean ± SD; ns, not significant (n=3). (Student’s Test).

Supplementary Figure 2 Total RNA isolated was subjected to Reverse Transcriptase-PCR analysis of uPAR-3’UTR expression, and *β2M* was used as a loading control (n = 3).

Supplementary Figure 3 Cells were tested for EGFR expression by FACS analysis after anti-miR146a treatment for 24h (n=3). Values are mean ± SD; ***p < 0.0001 (Student’s Test).

Supplementary Figure 4 Cellular growth counting the total number of cells 24 and 48 h after the initiation of the culture. (n = 3). In all the three cell lines evaluated the +3’UTR samples did not show any significant changes with respect to their KO counterparts, demonstrating that only the rescue of PLAUR expression is able to restore cell proliferation to control level.

Supplementary Figure 5 Whole Western Blot picture reporting uncropped bands.
