# Supplementary figures and images for: CRISPR/Cas9 uPAR Gene Knockout Results in Tumor Growth Inhibition, EGFR Downregulation and Induction of Stemness Markers in Melanoma and Colon Carcinoma Cell Lines
Source: Front Oncol. 2021 May 14;11:663225. doi: 10.3389/fonc.2021.663225 (PMC8163229; doi:10.3389/fonc.2021.663225)

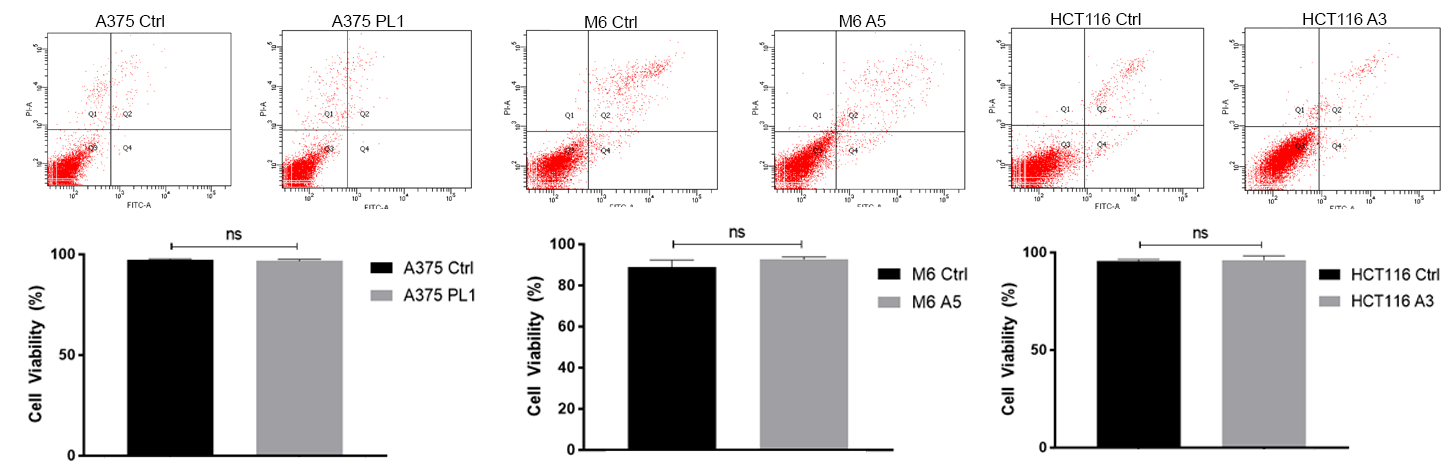

Supplement: Supplementary Figure 1 — Annexin V/PI assay on control and uPAR KO cells. Values are mean ± SD; ns, not significant (n=3). (Student’s Test). [file Image_1.tif]

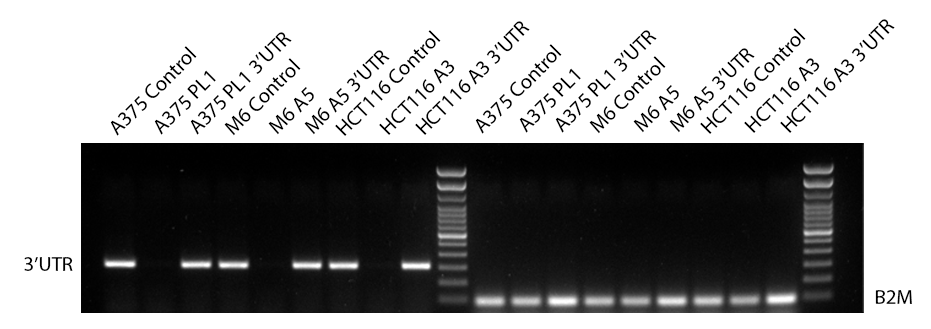

Supplement: Supplementary Figure 2 — Total RNA isolated was subjected to Reverse Transcriptase-PCR analysis of uPAR-3’UTR expression, and β2M was used as a loading control (n = 3). [file Image_2.tif]

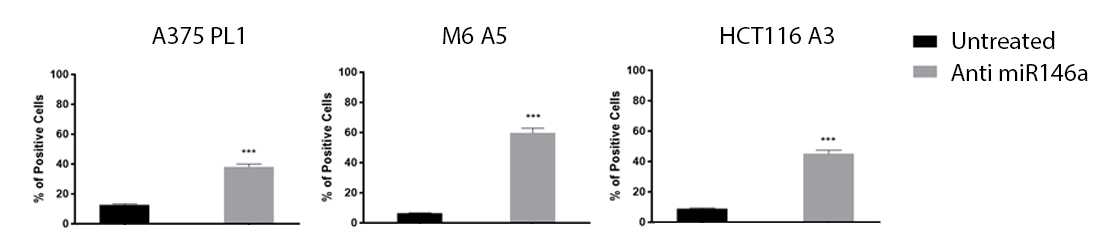

Supplement: Supplementary Figure 3 — Cells were tested for EGFR expression by FACS analysis after anti-miR146a treatment for 24h (n=3). Values are mean ± SD; ***p < 0.0001 (Student’s Test). [file Image_3.tif]

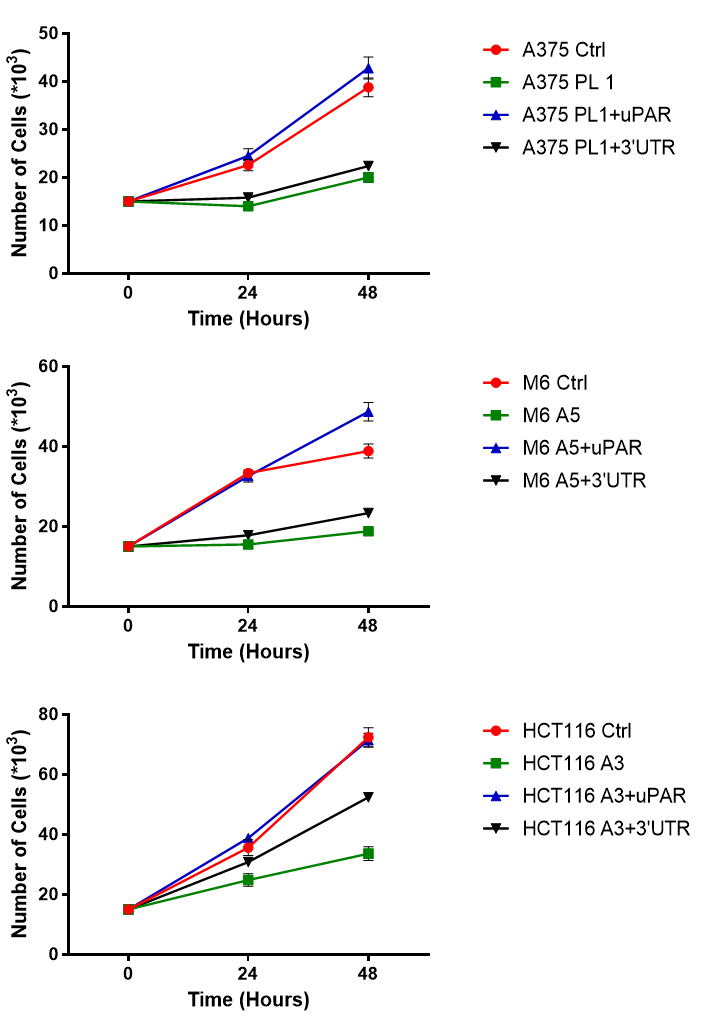

Supplement: Supplementary Figure 4 — Cellular growth counting the total number of cells 24 and 48 h after the initiation of the culture. (n = 3). In all the three cell lines evaluated the +3’UTR samples did not show any significant changes with respect to their KO counterparts, demonstrating that only the rescue of PLAUR expression is able to restore cell proliferation to control level. [file Image_4.tif]

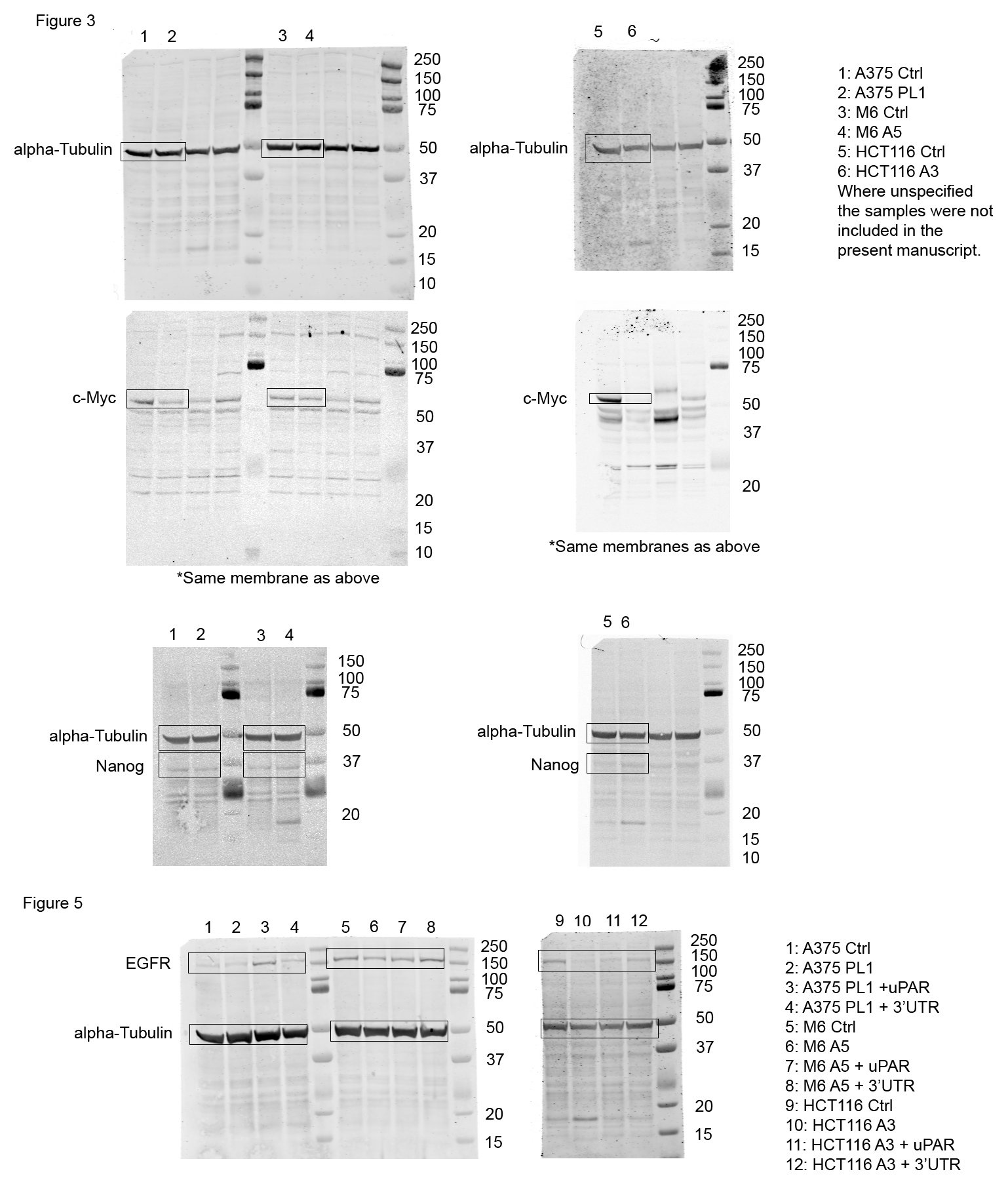

Supplement: Supplementary Figure 5 — Whole Western Blot picture reporting uncropped bands. [file Image_5.tif]
